# Care for Carers: an Investigation on Family Caregivers’ Needs, Tasks, and Experiences

**Published:** 2019-01-06

**Authors:** V Zavagli, M Raccichini, G Ercolani, L Franchini, S Varani, R Pannuti

**Affiliations:** 1 ANT Foundation, Italy

**Keywords:** caregiving, family caregiver, cancer, needs, oncological home-care, palliative home-care

## Abstract

Family caregivers have an essential active role in cancer patients assistance at home. They play a key role in the management of patients and provide some caregiving activities once provided only by professional caregivers. Often they are not adequately trained or prepared, however a systematic assessment of their needs is rarely practiced. For these reasons, this preliminary investigation was designed to better identify the needs and changes in the lifestyles of family caregivers of home cancer palliative care. Participants have completed a battery of self-report questionnaires, including the Caregiving Tasks Consequences and Needs Questionnaire (CaTCoN), that measures caregivers’ experiences (the extent of cancer caregiving tasks and consequences) and the caregivers’ needs, mainly concerning the interaction with the health care professionals. The results confirmed that cancer caregiving is burdensome. Large proportions of caregivers experienced substantial caregiving workload as well as a range of negative consequences, e.g. lack of time for social relations. Furthermore, considerable proportions of caregivers experienced problems or had unmet needs regarding the interaction with health care professionals. Prominent problematic aspects included the provision of enough information to the caregivers and attention to the caregivers’ well-being and feelings. The assessment of caregivers’ needs is a critical step for determining appropriate support services, providing high-quality care, achieving caregiver satisfaction, and decreasing caregiver burden. Findings of this investigation will certainly contribute to develop and publish Guidelines and to provide programmes and on-going education where caregivers feel supported in their role.

## I. INTRODUCTION

Cancer is a family disease and the World Health Organization has recommended approaching patients and their caregivers as a ‘unit of care’, focusing on the overall well-being of the patient-caregiver dyad rather than just on the patient.

In fact, the current health policy trend is to downsize acute-care hospitals and to transfer a greater portion of care at home, where family members form a substantial part of the care system. It leads an increased pressure mostly for family caregivers, bringing considerable responsibilities, needs and problems [1].

A “family caregiver” is considered anyone (parents, adult children, spouses…) who provides any type of physical and emotional care for an ill loved one at home [2]. Thus family cancer caregivers can be considered an extension of the health-care team, yet often they take on a new role for which they may not feel adequately prepared and are nervous or overwhelmed about what is expected of them. In fact, they find themselves having to perform new and unfamiliar tasks (giving medicines, assisting with meals, and performing medical and nursing procedures) and may experience a number of mixed emotions including anxiety, anger, and sadness [2].

Nowadays family caregivers are essential health team members: they play a key role in the management of patients with cancer and provide some caregiving activities once provided only by professional caregivers. Often they are not adequately trained or prepared and it is well known that caregiving to a family member with cancer might have health implications [3–9]. However limited research has investigated the psycho-physical disorders of home-cared cancer patients family caregivers and a systematic assessment of their needs is rarely practiced. In fact, evaluation is often informal and undocumented, making caregivers’ needs less “visible”.

Some studies indicate that caregivers’ unmet needs are multidimensional and that they affect caregivers’ health and well-being as well as the quality of care they provide to the patients [10–22].

Caregivers most frequently identify communication [10,14,16,19,21,22], information [10,18,19,21], assistance with personal care [11,12,16,17,19,20,22], support with technical daily tasks [11,18–20], respite care [11,13,15,19] and financial assistance [14,20,22] as areas where more support would be needed.

In fact, one of the greatest unmet needs was an open, effective, adequate, and comforting communication with healthcare professionals [10,14,16,19,21,22]. Caregivers said that the healthcare staff communicates without understanding and empathy [21], does not pay attention to the necessities of the patient’s family [19], and that does not have sufficient time to answer their questions [21]. In a study, the participants said that one of their greatest needs was effective and comforting communication with friends and acquaintances too [22]. According to them, friends should understand the conditions of patients and their caregivers, avoid too many questions about the patient’s condition, refrain from discussions of the future and the outcome of the disease, and avoid blame.

The need for more information was identified in several studies as well [10,18,19,21]. Caregivers wanted more information about the illness and how it would progress [19,20], often for the purpose of managing their lives and making decisions [10,22]. In addition, caregivers required more information about what to do at the time of the patient’s death. [10,14].

Practical unmet needs were discussed in almost all the studies [11,12,16–20,22]. The greatest necessities were support for care and assistance (e.g. help with the patient’s daily activities, symptom management, therapeutic drug monitoring, coordination for examinations and treatment, and equipment provision) and household tasks (e.g. housekeeping, caring for other family members, and other social responsibilities).

Among the psychosocial unmet needs were identified the lack of support with worries, fear of suffering and death, providing emotional support to the patient, and coping with an unpredictable future [10,15,17,22].

Moreover, most caregivers identified many negative reactions to caregiving, such as fatigue or weariness, depression, anger and sadness, financial stresses, and lack of time [14,15,20,22].

Given the interest of the theme and the literature data described above, this investigation was designed to better identify unmet needs and lifestyles’ changes of family caregivers of home cancer palliative care and subsequently correlate them with the patients’ functional status in order to investigate if caregivers’ needs change as the patients’ functional abilities change.

## II. METHODOLOGY

### Data collection has begun

Participants are enrolled in Italy among the caregivers of the patients assisted by the National Tumor Assistance (ANT) Foundation through its 20 oncological hospitals at home.

ANT is an Italian no-profit Foundation that provides since 1985 free medical, nursing, psychological and social home care and support for cancer patients through its 20 oncological hospitals at home in 9 Italian regions [23, 24]. ANT is one of the leading organizations in Italy in the field of palliative home care and pain management and since its foundation it has assisted more than 110.000 patients.

Till now data were collected from 87 family caregivers, but data collection is still in progress and the goal is to reach a sample of at least 200 caregivers during the coming months.

A summary of the characteristics of the subjects who took part in the investigation is presented in Table 1. The socio-demographic data of our sample confirm the characteristics already observed in the literature about cancer caregivers. In fact, this role is played mostly by women (70–80%) with an average age of 50–55 years [25].

Caregivers included in this study (I) are all living together with the patient; (II) are regularly providing care to their adult cancer patient at home since the time of diagnosis (by managing the symptoms/pain of the patient at home, giving personal care, supporting the patient in the house and hospital/bureaucratic settings and providing emotional support); (III) are not receiving any financial support for their caregiving work; and (IV) are 18 or older and played a key role in daily contact with physicians.

Participants filled out autonomously a battery of self-report questionnaires focusing on patient’s Index of Independence in Activities of Daily Living (i), patient’s Index of Independence in Instrumental Activities of Daily Living (ii) and Caregiving Tasks, Consequences and Needs (iii). They underwent tests during the home-care assistance provided by ANT Foundation and completed the first two questionnaires with reference to their patients’ condition.

- The Activities of Daily Living (ADL) [26] is a scale that contains a series of basic activities performed by individuals on a daily basis. It includes the fundamental skills necessary for independent living at home or in the community and it comprises the following areas: grooming/personal hygiene, dressing, toileting/continence, transferring/ambulating, and eating.
- The Instrumental Activities of Daily Living (IADL) [27] is a scale that contains a series of actions that are important to be able to live independently but are not necessarily required on a daily basis. It can be useful to determine with greater detail the level of assistance required by a sick person.
- The Cancer Caregiving Tasks, Consequences and Needs (CaTCoN) [28] is a 72-item questionnaire that measures cancer caregiving *tasks*, *consequences* and *needs* mainly concerning information from and communication and contact with health care professionals. The validity and reliability of the CaTCoN were evaluated by using psychometric analyses and were found to be satisfactory.

In addition, socio-demographic data were retrieved (sex, age, marital status, education level etc.).

The investigation received a formal approval by the Area Vasta Emilia Centro Research Ethical Committee of Emilia-Romagna Region (CE-AVEC).

Participants gave informed written consent for participation to the investigation, data analysis, and data publication.

## III. RESULTS

All analyses were conducted using SPSS 24.0 for Windows. Since the data collection is still in progress, these are preliminary results.

These preliminary results concern only the CaTCoN questionnaire.

For the total sample of caregivers, frequencies, mean and standard deviation scores for each CaTCoN item were calculated. The aspects perceived as most problematic by the total sample are pointed out following, using a cut-off of 30% of caregivers reporting problems or unmet needs [14]. The distribution of the caregivers’ answers to the most problematic items is also shown in Table 2.

### Caregiving tasks

Large proportions of caregivers experienced substantial caregiving workload: 91.2% of them provided some or a lot of practical help to the patient, 72.4% provided some or a lot of personal care, and 62.8% provided some or a lot of psychological support. Moreover, 51.7% of caregivers have felt to some or to a high degree responsible for keeping track of patient’s referrals and appointments for examinations and treatment, while 52.9% had spent a lot of time transporting the patient.

### Caregiving consequences

Regarding the negative social consequences caused by caregiving, 35.6% of caregivers reported that they had not enough time for friends and/or acquaintances.

### Caregiving needs

A great number of caregivers referred to problems or had unmet needs regarding the interaction with the health care professionals (HCPs).

Thirty percent (29.9%) of the caregivers reported that had received lacked information about how the health care system works in relation to treating cancer.

Of the caregivers, 60.9% reported that the HCPs in the hospitals rarely/never or only sometimes showed interest in whether they as caregivers have been able to handle the situation. More than half of the participants, 55.1% and 59.7% respectively, referred that the HCPs only rarely/never or sometimes paid attention to them and had shown interest in how they had been feeling.

Regarding provision of information, 32.2% of the caregivers reported that not enough time had been spent informing them and that often they had to ask the HCPs questions in order to get the information they needed (37.9%). Moreover, 32.2% of caregivers had to some degree lacked information about where to get help.

Finally, more than half of the caregivers reported that HCPs had not given them an unrealistically positive idea of the patient’s situation (71.3%) or have deprived them of hope (66.7%).

## IV. DISCUSSION

The main aim of the present investigation was to provide information about unmet needs and lifestyles’ changes of the family caregivers of cancer patients assisted at home.

Family caregivers are an invaluable part of healthcare teams whose needs remain unmet despite their active role in patient care. In fact, it is well known that family caregivers often give priority to their patient’s necessities, although they have multiple unmet needs that have a negative impact on their quality of life and consequently on the quality of care they provide to the patient.

The preliminary results confirmed that cancer caregiving is burdensome. Large proportions of caregivers experienced substantial caregiving workload related to practical help, emotional support, and transport. These findings are in agreement with previous findings [11,12,15,16–20,22].

Moreover, participants reported a range of negative consequences of being a caregiver, most commonly lack of time for social relations. Thus, in line with the literature [14,15,20,22], these preliminary results demonstrate that being a caregiver is demanding and has its costs.

Finally, this investigation shows that information is an important element of cancer care. In fact, large proportions of caregivers experienced problems or had unmet needs regarding the interaction with health care professionals. Prominent problematic aspects included the provision of enough information to the caregivers and attention to the caregivers’ well-being and feelings. These findings are in agreement with previous studies [10,18,19,21], showing that dissatisfaction with information is common to different countries. Therefore, hopefully, these results will lead the way for improving the information and the caregivers’ satisfaction with the interaction.

Contrary to some previous research [14,20,22], financial consequences did not appear to be a significant concern for the participants in this investigation. Probably this is due to the fact that in Italy palliative care is available and financed by the government and charity organizations.

Thereby, this study also highlights several directions for future research. The findings of this investigation can be viewed as guidance for healthcare policy planning and the design of interventions for improving quality of life in family caregivers who are high risk. In fact, many European countries are introducing policies to support effective programs to enhance active and healthy ageing [29, 30]. Moreover, some studies have found that integrated care services, closely oriented to the needs of patients/users, multidisciplinary, and anchored in community and home care settings, can benefit informal caregivers [31, 32].

Obviously, this is a preliminary investigation and limitations must be acknowledged. The transferability of the findings to other settings and populations is limited. Our investigation was restricted to family caregivers of palliative cancer patients assisted at home, the results cannot be generalized across all treatment phases.

## V. CONCLUSION

We strongly believe that the assessment of caregivers’ experiences (caregiving tasks and consequences) and needs, mainly concerning the interaction with the health care professionals, is a critical step for determining appropriate support services, providing high-quality care, achieving caregiver satisfaction, and decreasing caregiver burden. In fact, caregivers will feel their roles are validated and their needs become more visible. Moreover, the findings of investigations like these will allow implementing a series of personalized clinical and organizational actions aimed at intervening on the critical issues emerged (in particular, the theme of information).

This can constitute a basis for then develop and publish Guidelines and to provide programs and on-going education where caregivers feel supported in their role.

Furthermore, investigations of this kind can urge legislators to recognize the caregivers’ figure. In fact, there is not yet a common European law that protects and improves the role and status of the caregivers and the different countries have a different cultural approach to the issue.

## 





## Figures and Tables

**Table 1:** Study Population

		Caregivers
	N	87
	Age [yrs]	62.2 ± 12.9
	Gender (male/female)	33/54
	Caregiver role:	
-	husband or wife [%]	66.7
-	son or daughter [%]	23
-	other relatives [%]	10.3
	Caregiving duration [mos]	
-	> 6 mos [%]	92
-	< 6 mos [%]	8
	Employment [%]	39
	Years of Education:	
-	0–8 yrs [%]	46
-	13 yrs [%]	36.8
-	≥ 16 yrs [%]	17.2

**Table 2:** Frequencies (%) and mean scores of responses regarding care-giving tasks and consequences (n = 87 caregivers)

CaTCoN single items	Frequencies%					Mean	S.D.
**1. To what extent have you had to provide:**	*None*	*A little*	*Some*	*A lot*	*Don’t Know/not relevant*		
**1a. Practical help to the patient?**	1.1	5.7	35.6	56.3	1.1	2.51	0.680
**1b. Personal care to the patient?**	9.2	16.1	29.9	42.5	2.3	2.13	1.021
**1c. Psychological support to the patient?**	7.0	27.9	37.2	25.6	2.3	1.88	.951
	*No, not at all*	*To a low degree*	*To some degree*	*To a high degree*	*Don’t Know/not relevant*		
**2. Have you felt that you have been partially responsible for keeping track of whether the patient has been referred and called for examinations and treatments quickly and correctly?**	14.9	19.5	27.6	33.3	4.6	1.93	1.149
**3. Have you felt that you have had too much responsibility in relation to home care (personal care, medications, etc.)?**	21.8	24.1	33.3	18.4	2.3	1.55	1.097
	*No, not at all*	*Yes, a little*	*Yes, some*	*Yes, a lot*	*Don’t Know/not relevant*		
**4. Have you spent time transporting the patient?**	9.2	10.3	26.4	52.9	1.1	2.26	.994
**6. Has the patient’s cancer disease:**							
**6c. Meant that you have not had enough time for (the rest of) your family?**	17.2	23.0	26.4	23.0	10.3	1.86	1.250
**6d. Meant that you have not had enough time for (the rest of) your friends/acquaintances?**	9.2	24.1	29.9	35.6	1.1	1.95	1.011
	*Always/almost always*	*Mostly*	*Only sometimes*	*Rarely/Never*	*Don’t Know/not relevant*		
**10. Have the health care professionals paid attention to you?**	23	17.2	26.4	28.7	4.6	1.48	1.256
**11. Have the health care professionals shown interest in how you have been feeling?**	16.1	18.4	21.8	37.9	5.7	1.30	1.286
**14. We would like to know whether you have lacked (more) information about different areas. Have you as a caregiver:**	*No, not at all*	*To a low degree*	*To some degree*	*To a high degree*	*Don’t Know/not relevant*		
**14a. Lacked information about how the health care system works in relation to treating cancer?**	14.9	32.2	29.9	21.8	1.1	1.62	1.026
**14b. Lacked information about how long one has to wait at different times in the process?**	16.1	40.2	25.3	14.9	3.4	1.49	1.044
**14e. Lacked information about the illness and its course?**	9.2	35.6	37.9	14.9	2.3	1.66	.925
	*Always/almost always*	*Mostly*	*Only sometimes*	*Rarely/Never*	*Don’t Know/not relevant*		
**15. Have you received adequate assistance when you telephoned health care professionals to ask questions?**	10.8	30.8	20	36.9	1.5	1.88	1.083
	*No, not at all*	*To a low degree*	*To some degree*	*To a high degree*	*Don’t Know/not relevant*		
**18. Have you felt that the health care professionals have given you an unrealistically positive idea of the patient’s situation?**	71.3	16.1	4.6	2.3	5.7	.55	1.086
**19. Have you felt that the health care professionals have deprived you of hope?**	66.7	10.3	8.0	9.2	5.7	.77	1.264
**21. Do you think enough time has been spent informing caregivers?**	16.1	32.2	41.4	9.2	1.1	1.47	.913
	*Always/almost always*	*Mostly*	*Only sometimes*	*Rarely/Never*	*Don’t Know/not relevant*		
**22. Have you had to ask the health care professionals questions in order to get the information you have needed?**	11.5	37.9	47.1	3.4	-	1.57	.741
**23. Have you lacked being given information from the health care professionals, without having to ask for it yourself?**	9.3	22.1	39.5	29.1	-	1.12	.938
**29. Have the health care professionals in the hospitals shown interest in whether you as a caregiver have been able to handle the situation?**	9.2	27.6	25.3	35.6	2.3	1.17	1.091
**30. Have the health care professionals in the hospitals noticed and reacted to signals from you, if you have not been doing well?**	5.7	3.4	14.9	56.3	19.5	1.17	1.608
	*No, not at all*	*To a low degree*	*To some degree*	*To a high degree*	*Don’t Know/not relevant*		
**33. Have you lacked information about where to get help as a caregiver?**	31.0	32.2	17.2	8.0	11.5	1.37	1.313
