# Supplementary material for: Care for Carers: an Investigation on Family Caregivers’ Needs, Tasks, and Experiences
Source: Transl Med UniSa. 2019 Jan 6;19:54–9. (PMC6581485)
Supplement: Supplementary file 1 [file TM-19-054-s001.doc]

|  | **Caregivers** |
| --- | --- |
| N | 87 |
| Age [yrs] | 62.2 ± 12.9 |
| Gender (male/female) | 33/54 |
| Caregiver role: |  |
| - husband or wife [%] | 66.7 |
| - son or daughter [%] | 23 |
| - other relatives [%] | 10.3 |
| Caregiving duration [mos]   - > 6 mos [%] - < 6 mos [%]   Employment [%]  Years of Education: | 92  8  39 |
| - 0-8 yrs [%] | 46 |
| - 13 yrs [%] | 36.8 |
| - ≥ 16 yrs [%] | 17.2 |

Table 1 - Study Population
