# Supplementary material for: Care for Carers: an Investigation on Family Caregivers’ Needs, Tasks, and Experiences
Source: Transl Med UniSa. 2019 Jan 6;19:54–9. (PMC6581485)
Supplement: Supplementary file 2 [file TM-19-054-s002.doc]

| **CaTCoN single items** | **Frequencies%** | | | | | **Mean** | **S.D.** |
| --- | --- | --- | --- | --- | --- | --- | --- |
| **1. To what extent have you had to provide:** | *None* | *A little* | *Some* | *A lot* | *Don’t Know/not relevant* |  |  |
| **1a. Practical help to the patient?** | 1.1 | 5.7 | 35.6 | 56.3 | 1.1 | 2.51 | 0.680 |
| **1b. Personal care to the patient?** | 9.2 | 16.1 | 29.9 | 42.5 | 2.3 | 2.13 | 1.021 |
| **1c. Psychological support to the patient?** | 7.0 | 27.9 | 37.2 | 25.6 | 2.3 | 1.88 | .951 |
|  | *No, not at all* | *To a low degree* | *To some degree* | *To a high degree* | *Don’t Know/not relevant* |  |  |
| **2. Have you felt that you have been partially responsible for keeping track of whether the patient has been referred and called for examinations and treatments quickly and correctly?** | 14.9 | 19.5 | 27.6 | 33.3 | 4.6 | 1.93 | 1.149 |
| **3. Have you felt that you have had too much responsibility in relation to home care (personal care, medications, etc.)?** | 21.8 | 24.1 | 33.3 | 18.4 | 2.3 | 1.55 | 1.097 |
|  | *No, not at all* | *Yes, a little* | *Yes, some* | *Yes, a lot* | *Don’t Know/not relevant* |  |  |
| **4. Have you spent time transporting the patient?** | 9.2 | 10.3 | 26.4 | 52.9 | 1.1 | 2.26 | .994 |
| **6. Has the patient's cancer disease:** |  |  |  |  |  |  |  |
| **6c. Meant that you have not had enough time for (the rest of) your family?** | 17.2 | 23.0 | 26.4 | 23.0 | 10.3 | 1.86 | 1.250 |
| **6d. Meant that you have not had enough time for (the rest of) your friends/acquaintances?** | 9.2 | 24.1 | 29.9 | 35.6 | 1.1 | 1.95 | 1.011 |
|  | *Always/almost always* | *Mostly* | *Only sometimes* | *Rarely/Never* | *Don’t Know/not relevant* |  |  |
| **10. Have the health care professionals paid attention to you?** | 23 | 17.2 | 26.4 | 28.7 | 4.6 | 1.48 | 1.256 |
| **11. Have the health care professionals shown interest in how you have been feeling?** | 16.1 | 18.4 | 21.8 | 37.9 | 5.7 | 1.30 | 1.286 |
| **14. We would like to know whether you have lacked (more) information about different areas. Have you as a caregiver:** | *No, not at all* | *To a low degree* | *To some degree* | *To a high degree* | *Don’t Know/not relevant* |  |  |
| **14a. Lacked information about how the health care system works in relation to treating cancer?** | 14.9 | 32.2 | 29.9 | 21.8 | 1.1 | 1.62 | 1.026 |
| **14b. Lacked information about how long one has to wait at different times in the process?** | 16.1 | 40.2 | 25.3 | 14.9 | 3.4 | 1.49 | 1.044 |
| **14e. Lacked information about the illness and its course?** | 9.2 | 35.6 | 37.9 | 14.9 | 2.3 | 1.66 | .925 |
|  | *Always/almost always* | *Mostly* | *Only sometimes* | *Rarely/Never* | *Don’t Know/not relevant* |  |  |
| **15. Have you received adequate assistance when you telephoned health care professionals to ask questions?** | 10.8 | 30.8 | 20 | 36.9 | 1.5 | 1.88 | 1.083 |
|  | *No, not at all* | *To a low degree* | *To some degree* | *To a high degree* | *Don’t Know/not relevant* |  |  |
| **18. Have you felt that the health care professionals have given you an unrealistically positive idea of the patient’s situation?** | 71.3 | 16.1 | 4.6 | 2.3 | 5.7 | .55 | 1.086 |
| **19. Have you felt that the health care professionals have deprived you of hope?** | 66.7 | 10.3 | 8.0 | 9.2 | 5.7 | .77 | 1.264 |
| **21. Do you think enough time has been spent informing caregivers?** | 16.1 | 32.2 | 41.4 | 9.2 | 1.1 | 1.47 | .913 |
|  | *Always/almost always* | *Mostly* | *Only sometimes* | *Rarely/Never* | *Don’t Know/not relevant* |  |  |
| **22. Have you had to ask the health care professionals questions in order to get the information you have needed?** | 11.5 | 37.9 | 47.1 | 3.4 | - | 1.57 | .741 |
| **23. Have you lacked being given information from the health care professionals, without having to ask for it yourself?** | 9.3 | 22.1 | 39.5 | 29.1 | - | 1.12 | .938 |
| **29. Have the health care professionals in the hospitals shown interest in whether you as a caregiver have been able to handle the situation?** | 9.2 | 27.6 | 25.3 | 35.6 | 2.3 | 1.17 | 1.091 |
| **30. Have the health care professionals in the hospitals noticed and reacted to signals from you, if you have not been doing well?** | 5.7 | 3.4 | 14.9 | 56.3 | 19.5 | 1.17 | 1.608 |
|  | *No, not at all* | *To a low degree* | *To some degree* | *To a high degree* | *Don’t Know/not relevant* |  |  |
| **33. Have you lacked information about where to get help as a caregiver?** | 31.0 | 32.2 | 17.2 | 8.0 | 11.5 | 1.37 | 1.313 |

Table 2 - Frequencies (%) and mean scores of responses regarding care-giving tasks and consequences (n = 87 caregivers)
